# Army Combat Fitness Test Relationships to Tactical Foot March Performance in Reserve Officers’ Training Corps Cadets

**DOI:** 10.3390/biology12030477

**Published:** 2023-03-21

**Authors:** Kevin L. Withrow, Daniela A. Rubin, J. Jay Dawes, Robin M. Orr, Scott K. Lynn, Robert G. Lockie

**Affiliations:** 1 16th Combat Aviation Brigade, Holistic Health and Fitness, Joint Base Lewis-McChord, WA 98433, USA; 2 Center for Sport Performance, Department of Kinesiology, California State University, Fullerton, CA 92831, USArlockie@fullerton.edu (R.G.L.); 3 Tactical Fitness and Nutrition Lab, School of Kinesiology, Applied Health and Recreation, Oklahoma State University, Stillwater, OK 74078, USA; 4 Tactical Research Unit, Bond University, Robina, QLD 4229, Australia

**Keywords:** ACFT, Army, combat fitness, leg tuck, military, ROTC, ruck march, soldier, sprint-drag-carry, 2-mile run

## Abstract

**Simple Summary:**

This study analyzed the relationships between the Army Combat Fitness Test (ACFT; deadlift, standing power throw, hand release push-up, sprint-drag-carry, leg tuck/plank, 2-mile run) and a 6.44-km tactical foot march (TFM) in Reserve Officers’ Training Corps cadets. Data from 29 cadets were analyzed. Cadets carried a 15.88-kg rucksack, fighting load carrier, 3-L hydration pack, and replica M4 carbine during the TFM. Partial correlations controlling for sex determined ACFT and TFM relationships. ACFT total score, leg tuck, 2-mile run, and sprint-drag-carry were associated with the TFM. Aerobic and anaerobic capacity, and upper body/trunk strength emerged as fitness components related to cadet TFM performance.

**Abstract:**

The Army Combat Fitness Test (ACFT), consisting of deadlift, standing power throw, hand release push-up, sprint-drag-carry, leg tuck or plank, and 2-mile run, is the United States Army’s new fitness test. The ACFT is designed to measure multiple fitness components required to perform combat tasks. One critical task is the tactical foot march (TFM), where soldiers cover long distances while carrying loads comprised of mission-essential equipment. As the ACFT is meant to predict soldier task performance, determining the relationships between the ACFT and the TFM is important. Data from 29 cadets (♂ = 20, ♀ = 9) from one university Reserve Officers’ Training Corps program were analyzed. The ACFT was recorded in raw and scaled scores. The TFM was performed over 6.44 km, with time recorded. Cadets carried a 15.88-kg rucksack, fighting load carrier, 3-L hydration pack, and replica M4 carbine. Independent samples t-tests evaluated ACFT and TFM between-sex differences. Partial correlations, controlling for sex, determined ACFT event and TFM relationships. Male cadets outperformed females in all ACFT tasks (*p* ≤ 0.039), except the push-up. ACFT total score, leg tuck, 2-mile run, and sprint-drag-carry showed large correlations with the TFM (*r* = ±0.463–0.531, *p* ≤ 0.026). Aerobic and anaerobic capacity and upper body/trunk strength were important fitness components for cadet TFM performance.

## 1. Introduction

The United States (US) Army Reserve Officers’ Training Corps (ROTC) is a two- or four-year program for university students to learn leadership and basic military skills in preparation for service as commissioned officers in the US Army upon graduating. Most Army officers are commissioned through ROTC; accordingly, it is an important initial training program for officers, as it is their first exposure to the Army. ROTC programs are located on many university campuses throughout the US [1]. University students who decide to join ROTC are known as cadets. Cadets are categorized by military science, or “MS” level: freshmen are “MSI”, sophomores “MSII”, juniors “MSIII”, and seniors “MSIV” [2]. Cadets take “military science” classes offered through the ROTC program at their school, from which they receive elective credit towards their degree [2]. Cadets also must attend summer training programs specific to their MS level, located at Fort Knox, KY [2]. During the academic semester, cadets generally participate in physical fitness training several days per week. Their physical fitness is assessed by the training cadre, and they must meet Army minimum standards before commissioning as officers.

Measuring the physical fitness of military personnel is vital because it can gauge the readiness of soldiers to perform in a combat environment [3,4]. As such, any occupationally specific physical fitness test used for active-duty personnel should be a valid predictor of ability to perform combat tasks. Indeed, the physical fitness required for combat is unique compared to other domains (e.g., sport, employment, general health) [3,4]. The US Army has recently replaced its decades old physical fitness test (Army Physical Fitness Test; APFT) with a new test that should better evaluate the physical fitness necessitated by combat; the Army Combat Fitness Test (ACFT). The APFT consisted of 2-min push-ups, 2-min sit-ups, and a 2-mile run. The APFT measured muscular and aerobic endurance, but neglected other important domains of fitness, including strength, power, and anaerobic endurance [5]. Common soldier tasks are physically demanding and require a high level of fitness in multiple domains beyond endurance [4,6,7,8,9,10], and the APFT was found to poorly predict performance in soldier tasks [11]. This major drawback of the APFT was that it negatively affected the way the Army conducted physical fitness training. Because of the importance of APFT score on career advancement, many soldiers trained in ways that only prepared them for the endurance events of the APFT, which left them ill-prepared for the demands of combat and increased their injury risk [4,12,13,14]. The ACFT tests multiple components of fitness beyond endurance, and it was in part created to encourage soldiers to incorporate these components into their training so that they could perform better in their job-related tasks. The ACFT has six scored events worth 100 points each that are summed to create a total score out of 600, with a minimum passing score of 60 points in each event [15]. The original six events were the 3-repetition maximum deadlift (deadlift), standing power throw, hand-release push-ups (push-ups), sprint-drag-carry, leg tuck, and 2-mile (3.22 km) run [15]. These events were intended to measure strength, power, upper body muscular (strength) endurance, anaerobic capacity, upper body and ‘core’ (trunk) strength, and aerobic capacity, respectively [16].

Over the past few months, the Army made several changes to the ACFT. First, the plank was introduced as an alternative event to the leg tuck as many female soldiers lacked the upper body strength necessary to pass the leg tuck [17]. If soldiers failed the leg tuck, they were allowed to do the plank, but they could only receive the minimum passing score of 60 points if successful. Then, the Army allowed soldiers to choose between the leg tuck or plank with both events being equally graded. Finally, the leg tuck was replaced by the plank, and ACFT scoring standards were changed to a new scale based on sex and age [16]. These final changes were made in accordance with recommendations from a congressionally mandated Army-sponsored independent review of the ACFT [18]. The review by Hardison, et al. [19] found that the ACFT was a valid test of the fitness required for combat task performance, except for the leg tuck and plank. In addition to this, Hardison, et al. [19] documented that female soldiers were failing at a high rate, and that changes should be made to the scoring to lessen the impact the test had on females. Thus, the focus of the ACFT has shifted more to general fitness and less on combat readiness. Nevertheless, the ACFT still provides a measure of a range of different fitness qualities that are of importance to a ROTC cadet.

One essential soldier task that is a hallmark of military service and combat is the tactical foot march (TFM) [20,21,22,23]. Dismounted soldiers must often move on foot across long distances, over varying terrain, and under time constraints, while carrying a heavy load of over 45 kg [24,25] consisting of a rucksack, body armor, helmet, and weapon and arrive at their destination ready to engage with the enemy. Mission success often depends on speed and distance covered while carrying a load [20,21,22,23]. The combat arms professions (e.g., infantry) place an extremely high value on a soldier’s ability to perform the TFM. The TFM is practiced regularly as a part of the physical training plan in combat arms units. The frequency, duration, and intensity of TFM practice varies by Army unit, as it is ultimately at the discretion of commanders; however, the Army has published guidance on how to train for the TFM [3]. Units generally practice the TFM as a group in formation with platoon-size elements (approximately 30 soldiers) whereby the pace is set by the leader. Commanders will often assess individual performance at the beginning and end of the training block by conducting a TFM in which soldiers move individually at their maximum pace. TFM performance has been shown to be most affected by aerobic endurance and strength, and anaerobic capacity becomes relevant at higher intensities [21,22,23]. However, there is limited research on what physiological characteristics affect TFM performance. This is especially true when considering ROTC cadets, who may demonstrate different physiological characteristics when compared to individuals who enlist in the US Army without going to college first.

As the TFM is a critical soldier task, it is important to determine if the ACFT relates to TFM performance in ROTC cadets. Since the ACFT measures multiple components of fitness, it is essential to ascertain which of the six events particularly relate to TFM performance. More effective training methods for soldiers could be utilized if these relationships are known. Therefore, the purpose of this study was to investigate the relationships between ACFT total score and raw and scaled scores in each of the six events with TFM performance. It was hypothesized that ACFT total score would have a significant relationship with TFM performance. Additionally, it was hypothesized that the 2-mile run, deadlift, sprint-drag-carry, and leg tuck events would have the largest correlations with TFM performance. Research has shown that the fitness components intended to be measured by these events are the most important in predicting TFM performance [21,22,23,26].

## 2. Materials and Methods

### 2.1. Participants

Retrospective fitness test data for 29 cadets (13 MSI, 11 MSII, 5 MSIV) were obtained from one university ROTC program. This was a convenience sample that included 20 males (age: 20.15 ± 2.39 years of age, height: 1.73 ± 0.07 m, body mass: 75.61 ± 11.34 kg) and 9 females (age: 20.22 ± 3.27 years of age, height: 1.61 ± 0.07 m, body mass: 63.24 ± 11.61 kg). The data for this study were collected by ROTC cadre, released with consent, and consisted of cadet scores from a previously conducted ACFT and TFM completed as a part of the regular physical and physiological monitoring of cadets. All available data were used for this study. The exclusion criterion was data that were clearly incorrect. The California State University, Fullerton Institutional Review Board approved the use of the pre-existing data (HSR-20-21-101). This study also conformed to the Declaration of Helsinki [27].

### 2.2. Procedures

The ACFT and TFM testing were conducted between 0500 and 0700 (5:00 AM and 7:00 AM). The ACFT data were collected at one university in southern California, and the TFM data were collected at Joint Forces Training Base Los Alamitos in Los Alamitos, California. The ACFT was conducted eight days prior to the TFM. Prior to starting the ACFT and TFM, cadets performed the Army’s dynamic warm-up, known as the “preparation drill,” consisting of 10 body-weight exercises: bend and reach, rear lunge, high jumper, rower, squat bender, windmill, forward lunge, prone row, bent-leg body twist, and push-up [28].

### 2.3. Army Combat Fitness Test (ACFT)

The ACFT consisted of six events that assessed the strength, power, muscular endurance, anaerobic capacity, and aerobic endurance of the cadets. The events, in order, were the deadlift, standing power throw, push-ups, sprint-drag-carry, leg tuck or plank, and 2-mile run. The testing time did not exceed 120 min [15]. There was no required rest between events, except for a 10-min break after the leg tuck or plank [15]. Cadets rotated through each event in groups; therefore, they received some rest between events [15]. The test completed by the cadets in this study was scored according to the previous sex- and age-neutral ACFT scale and was conducted according to Army standards by the cadre at their respective university’s outdoor sports complex. Detailed procedures for each event can be found in Army Techniques Publication 7-22.01 [15]. Regardless, events are described hereafter.

The deadlift was performed using a hexagonal bar. Cadets lifted the bar off the ground by grasping the low handles and extending the hips and knees until standing erect, and then lowered the bar back to the ground with control. Cadets were to keep a neutral spine, their knees were not to go into valgus collapse, and they displayed no loss of balance. The event began with a 10-min warm-up with practice sets with a load of the cadet’s choosing. Then, the cadet selected a load to be graded for and performed three continuous repetitions. If they were not successful, or if they performed the movement outside of the prescribed technique, they were given another attempt with the opportunity to change the load. If they were successful on the first attempt, they were given the option to increase the load for a second attempt to obtain a higher score.

The standing power throw was performed with a 4.54 kg (10 lbs) medicine ball. The cadet grasped the ball and faced away from the start line with the heels at the line. The ball was thrown backwards over the head by first flexing the trunk, hips, and knees and then explosively extending them to jump up while flexing the shoulders. Cadets were allowed several preparatory movements of flexing and extending the trunk, hips, and knees. They were not allowed to fall or cross over the start line. Two attempts were given to throw the ball as far as possible. Results were measured to the nearest 0.1 m.

The push-up was a 2-min timed event where cadets did as many repetitions as possible. They began on the ground in the prone position with the hands just lateral to the shoulders and feet together or slightly apart with ankles dorsiflexed. They pushed the body off the ground by extending the elbows and horizontally adducting the shoulders and then lowered their body back to the ground. Upon reaching the ground, they fully extended the elbows so that the hands were off the ground and the arms were straight and perpendicular to the trunk, and then immediately brought the hands back to the starting position. The shoulders, hips, and knees had to move up and down as a single unit. Any rest was only to be taken in the up position.

The sprint-drag-carry was a timed 250-m shuttle event on a 25-m lane consisting of five 50-m shuttles. The first shuttle was a sprint, the second a drag, the third a lateral shuffle, the fourth a carry, and the fifth was another sprint. The cadets started in the prone position, then got up and sprinted 25 m, touched the line with the foot and hand, changed direction, and sprinted back to the start. The cadets then grabbed the handles of a 40.82-kg (90 lbs) sled and ran backwards 25 m, dragging the sled across the line, then turned around and dragged it back to the start. Next, the cadets laterally shuffled 25 m, touched the line with the foot and hand, changed direction, and laterally shuffled back to the start, leading with the opposite foot. Then, the cadets picked up two 18.14-kg (40 lbs) kettlebells and ran 25 m, touched the line with the foot, and ran back to the start. Finally, the cadets sprinted 25 m, touched the line with the hand and foot, and sprinted back to the start. Cadets attempted to complete this as fast as possible. Time was recorded with a stopwatch by the grader to the nearest 1 s.

Cadets began the leg tuck by hanging from a pull-up bar, perpendicular to the bar, with arms straight, feet off the ground, and legs not crossed. To perform a repetition, they flexed their elbows, trunk, hips and knees to raise the knees so that they touched the elbows, and then returned to the start position. If the cadet did not touch both elbows with both knees, or they did not fully return to the start position, the repetition was not counted. Rest was allowed only in the start position. Cadets completed as many repetitions as possible.

Some cadets chose to be graded on the plank in place of the leg tuck. To attain a plank position, cadets began by lying prone on the ground and lifting both knees off the ground and moving the hips into a straight line with the legs, shoulders, and head. The elbows were aligned under the shoulders, together with the forearms on the ground forming a triangle, with the hands up to a fist’s width apart and not interlocked, and the feet on the ground and up to hip width apart. Cadets attempted to maintain this position for as long as possible while being timed, with a maximum time of 4:20 min:seconds. The test was terminated if the cadet touched the floor with any part of the body other than the feet, forearms, or hands; raised a foot or hand off the floor; or failed to maintain a straight-line position from head to feet. Time was recorded with a stopwatch by the grader to the nearest 1 s.

The 2-mile run was a timed event where cadets ran two miles as fast as possible at their own pace. They ran a flat 2-mile loop on asphalt streets on their university campus. They were allowed to walk or stop, but they were not allowed to be physically helped in any way or to leave the instructed route. Time was recorded to the nearest 1 s with a stopwatch by the grader at the start and finish, which were the same point.

### 2.4. Tactical Foot March (TFM)

As stated, the TFM was conducted at Joint Forces Training Base Los Alamitos in Los Alamitos, California. The distance was 6.44 km (4 mi), and an Army issued rucksack with a 15.88 kg (35 lbs)-load was worn by all cadets. Cadets also wore an Army issued fighting load carrier (vest with pouch attachments) and a 3-L water hydration pack, and carried by hand a replica M4 carbine. The route was on a dirt trail with flat terrain. Cadets all started at the same time and were instructed to walk or run as fast as possible at their own pace to complete the route. They were allowed to pace each other, but not to physically help each other in any way. Time was recorded with a stopwatch to the nearest 1 s by the grader at the start and finish, which were the same point.

### 2.5. Statistical Analysis

All statistical analyses were conducted using Statistics Package for Social Sciences (Version 28, IBM Corporation, New York, NY, USA). Descriptive data (mean ± standard deviation (SD)) were calculated for all variables. Normality of the data was assessed using the Kolmogorov–Smirnov test, and any outliers were treated with a Winsorization method [29,30,31]. Statistical significance for all analyses was set a priori at *p* ≤ 0.05. Any differences between male and female cadets were investigated with independent-samples t tests. Males have been shown to outperform females in military fitness tests [19,32,33,34,35], so it was important to confirm any between-sex differences as this would indicate the need to control for sex in the correlation analyses. Levene’s test for equality of variances was conducted to determine whether equal variances were to be assumed or not assumed. Effect sizes (*d*) were also calculated for the between-sex comparisons, where the difference between the means was divided by the pooled SD [36]. A *d* less than 0.2 was considered a trivial effect, 0.2 to 0.6 a small effect, 0.6 to 1.2 a moderate effect, 1.2 to 2.0 a large effect, 2.0 to 4.0 a very large effect, and 4.0 and above an extremely large effect [37].

For the second part of the analysis, partial correlations controlling for sex were calculated to investigate the relationships between ACFT total score, scores in each of the six ACFT events (both raw and scaled scores), and TFM performance [38,39,40]. The plank was excluded from any analyses, as only four cadets chose the plank over the leg tuck. Therefore, the leg tuck sample size was 25 (18 males, 7 females). One cadet did not complete the 2MR, so the sample size was 28 for this test (19 males, 9 females). All other variables had a sample size of 29 (20 males, 9 females). The correlation strength was designated as: 0 to 0.3 and 0 to −0.3, small; 0.31 to 0.49 and −0.31 to −0.49, moderate; 0.5 to 0.69 and −0.5 to −0.69, large; 0.7 to 0.89 and −0.7 to −0.89, very large; and 0.9 to 1 and −0.9 to −1, near perfect relationship [41].

## 3. Results

The data for raw scores of the six ACFT events and TFM time for the overall group, males, and females, are shown in Table 1. Males performed significantly better than females in the deadlift, standing power throw, sprint-drag-carry, leg tuck, and 2-mile run, with moderate-to-large effects. There were no significant differences in score between males and females for the push-up and TFM. The data for the scaled scores of the six ACFT events and ACFT total score are shown in Table 2. Males scored significantly higher than females in the deadlift, standing power throw, sprint-drag-carry, leg tuck, 2-mile run, and total score, also with moderate-to-large effects. There were no significant between-sex differences in score between males and females for the push-up.

The between-sex comparisons confirmed the need to control for sex in the correlation analyses. The partial correlation data for the TFM and raw scores for each of the six ACFT events are shown in Table 3. The 2-mile run and sprint-drag-carry showed large, significant positive correlations with the TFM, indicating that faster times in the two events related to a faster TFM time. The leg tuck showed a large, significant negative correlation with the TFM, indicating that more leg tuck repetitions related to a faster TFM time. There were no significant correlations between TFM and deadlift, standing power throw, and push-ups. The data for partial correlations between the TFM and scaled scores for each of the six ACFT events and ACFT total score are shown in Table 4. There were large, significant negative correlations between TFM and leg tuck, 2-mile run, and total score. There was a moderate, significant negative correlation between TFM and sprint-drag-carry. Higher scores in each of the events and the total ACFT score related to a faster TFM time. There were no significant relationships between the TFM and deadlift, standing power throw, or push-ups.

## 4. Discussion

This study examined the relationship between ACFT and TFM performance of cadets in one university US Army ROTC program. Firstly, male cadets performed significantly better compared to female cadets in every ACFT assessment, except push-ups and TFM. With regard to the ACFT and TFM correlation analyses, it was hypothesized that the ACFT total score and the deadlift, sprint-drag-carry, leg tuck, and 2-mile run events would be significantly related to the TFM. This hypothesis was supported in part by the findings of this study. The sprint-drag-carry, leg tuck, and 2-mile run showed relationships with a large degree of strength with the TFM. These results suggest that aerobic endurance, upper body and trunk strength, and anaerobic capacity may be important components of fitness necessary for performance in the TFM. These results support previous research in military personnel that suggest soldiers require sufficient fitness in multiple domains to successfully complete their required job tasks [4,6,7,8,9,10]. The present results also suggest that the ACFT measured these different fitness components, likely making the test valid and useful for measuring the fitness necessitated by combat, as the TFM is an essential combat task for soldiers [20,21,22,23].

Regarding the between-sex comparisons, the results of this study showed that male ROTC cadets significantly outperformed females, with moderate-to-large effects, in ACFT total score and every event except the push-ups. Although this study had a relatively small sample, the current data could have implications for the new ACFT sex-based scoring standards, which might mask physiological differences between the sexes affecting physical performance. Female soldiers with ACFT-scaled scores equal to males may have weaker raw scores than those males, which could indicate some limitations in tasks requiring the qualities measured in those tests (e.g., female soldiers may have equivalent sex-scaled deadlift scores with male soldiers, but a lower deadlift load, which indicates lesser strength). These findings also support previous research that has indicated female military personnel may require specific fitness training to reduce performance gaps and injury risk compared to their male counterparts [9,33,42,43] and the trend of males generally scoring higher than females in military fitness testing [32,33,34,35]. Data from the US Army also shows a gap in performance between the sexes [19]. This gap in performance is partly a contributing factor to the Army changing the ACFT to sex- and age-based scoring standards [18].

It should be noted that this change is somewhat contrary to the original logic behind why the ACFT was initially implemented. Indeed, the original aspirations for the ACFT was that it was supposed to be a fitness test designed to measure the components of fitness necessary for combat, regardless of sex or age [44]. This was because the arduous tasks of combat will not change for a soldier because of their sex or age. As previously noted, and given the data from this study and previous research [19,32,33,34,35], switching to a scoring system based on sex and age may serve to hide deficiencies in fitness that can hinder job performance. Inadequate fitness can put soldiers at greater risk, as combat tactics are reliant on the assumption of every soldier on a team being able to perform the tasks required of their job. The long-term impact of the ACFT changes requires further research. Nonetheless, the results from this study further highlight the need for female soldiers to have a specific physical training program that can target any fitness deficiencies compared to males [9,33,42,43] as there are sex differences in biomechanical, musculoskeletal, and physiological characteristics [45]. Furthermore, although further analysis is required, potentially hiding any sex (or age) differences via sex- and age-based scoring standards may deter the development of sound physical training programs for personnel, which could potentially harm Army readiness.

TFM performance is highly dependent on aerobic endurance [21,22,23,46], which partially explains why there was a large correlation with the 2-mile run. The 2-mile run provides a valid measure of aerobic capacity [47,48,49]. The cadets in this study, although a small sample from one school, did not perform well on the 2-mile run, with a mean time of approximately 17:35 min:seconds or 74 out of 100 points by ACFT scoring standards. Further, the cadets also did not perform well on the TFM, with a mean time of approximately 65 min or a 5.94 km/h (3.69 mi/h) pace. The Army minimum standard pace for a 19.31-km (12 mi) TFM with a 15.88-kg (35 lbs) load is 6.44 km/h (4 mi/h) or three hours [20]. Considering the TFM performed by the cadets was one-third that distance, on flat terrain, and with an equal load, the pace of the ROTC cadets would need to improve to meet minimum US Army standards. This could be due to cadet lack of experience, as TFM performance requires skill (e.g., pacing strategy) that has been shown to improve with task-specific training [22,26]. Interestingly, there was no significant between-sex differences in TFM performance. Regardless of sex, all the cadets likely need to improve their TFM performance. In addition to skill improvements [22,26], increasing aerobic endurance, reflected by lower 2-mile run times, should benefit TFM pace.

Anaerobic capacity becomes important to the TFM with increased intensity (e.g., load, pace, terrain) [23]. However, as previously stated, the cadets’ TFM pace and load was not performed at a high intensity relative to Army standards. Nevertheless, scaled and raw scores showed large and moderate correlations, respectively, with the TFM. The sprint-drag-carry was designed to provide a measure of anaerobic capacity within the ACFT [11,50]. Given the relationship with the sprint-drag-carry, the data from the current research suggested that the load and pace combination of the TFM was performed at a high enough intensity for the cadets, indicating a potential contribution from anaerobic capacity. It should be noted that the cadets demonstrated a relatively average performance on the sprint-drag-carry, with a mean score of approximately 78 out of 100 points or a raw time of 2:07 min:seconds. Hence, the cadets from this study could benefit from specific anaerobic training. Further, developing anaerobic capacity, in addition to other important fitness components (i.e., aerobic endurance, upper body and trunk strength) could allow the cadets to sustain higher TFM intensities. This should be confirmed via further research.

Recent research on the leg tuck performance in firefighters demonstrated that sex, pull-ups, and push-ups predicted 66.8% of the variance in the number of repetitions performed, with pull-ups being the largest contributor [51]. Considering this, work by Robinson et al. [46] and Orr et al. [52] found that, of all the strength measures assessed (deadlift, squat, bench press, and loaded pull ups), pull-ups was the strength metric that had the strongest relationship to load carriage performance in specialist police covering 5 km with a 25-kg pack (*r* = −0.452, *p* < 0.05) and 5 km with a 40-kg load (*r* = −0.356, *p* < 0.05), respectively. Given the actions required in the leg tuck, it could also be surmised that trunk strength is an important contributor to this movement [1]. When one considers how upper body and trunk strength have been found to be important in TFM performance [22,53], this provides some indication as to why the leg tuck showed a strong correlation with the TFM in the ROTC cadets from this study. During the TFM, the upper body musculature assumes much of the rucksack load and must work to stabilize the trunk [22]. In the present study, cadets achieved a mean score of approximately 80 out of 100 points for the leg tuck, or 10 repetitions. This suggests that their upper body and trunk strength could be improved to allow for a higher load tolerance and reduced relative intensity in the TFM, thus potentially increasing performance. Nonetheless, it is notable that the leg tuck related to TFM performance, especially considering that it has just been removed from the ACFT [18].

As previously acknowledged, the US Army recently replaced the leg tuck with the plank exercise in the ACFT [18]. Hardison et al. [19] suggested that the leg tuck did not have any relationship to combat task performance. Because female soldiers were the primary group failing the leg tuck (more than 70% of enlisted women), Hardison et al. [19] further stated that the leg tuck did not accurately measure their trunk strength because it also required upper body strength. This statement is partially supported by Lockie et al. [51], who documented the significant relationship between the leg tucks and pull-ups (*r* = 0.790, *p* < 0.001) in firefighters. Lockie et al. [51], also showed large sex difference in leg tuck performance between male (12.64 ± 5.49 repetitions) and female (5.90 ± 5.01 repetitions) firefighters. However, data from the present study show that performance in the leg tuck was strongly related with performance in the TFM, an essential combat task. Additionally, the original purpose of the leg tuck, as stated by the Army, was to measure upper body and core (trunk) strength [15]. Upper body strength is essential to the performance of soldier tasks that involve climbing, such as getting over a wall or up a rope [3,7,10]. By replacing the leg tuck with the plank, the Army may have lessened its ability to measure upper body strength within the ACFT. Furthermore, there is no current research that shows the plank has a relationship to combat performance, which was also stated in the independent analysis [19]. The US Army and command staff will need to reconcile this issue within the new ACFT with further research, and how replacing the leg tuck with the plank tests could influence soldier fitness and readiness for combat.

The US Army originally introduced the ACFT to encourage a better fitness training culture and to measure the multiple fitness components necessary for combat [3]. The overall ACFT score provides an indication of the total fitness of the cadet given the contributions of the different tests to the final score. Within the context of the cadet sample, the results of this study showed that the ACFT total score had a large correlation with TFM performance. This suggested that the ACFT measured multiple components of fitness that are potentially important for TFM performance. Nonetheless, the cadets had a mean total score of approximately 458 out of 600 points, indicating a need for improvement. Three of the six events in the ACFT (leg tuck, sprint-drag-carry, and 2-mile run) correlated to the TFM in this sample, which provides support for the association between the total ACFT scores and the TFM.

It was hypothesized that the deadlift would have a large correlation with the TFM, as the deadlift measures maximal lower body strength [15,54,55]. Following a review of the literature, Orr et al. [56] acknowledged that lower-body strength can be important for load carriage tasks such as the TFM. However, the results of this study did not show a significant relationship between the deadlift and TFM in this cadet sample. It could be that cadets had not yet developed their strength enough to be translated to the TFM, as their mean score for the deadlift was approximately 77 out of 100 points or 107 kg. Additionally, the 15.88 kg (35 lbs) load carried in the TFM that cadets completed may not have been heavy enough to create a need for cadets to express their maximal strength. The typical load carried by soldiers ranges from 15 to 57 kg [21,22,23], and maximal strength becomes important with heavier loads [57]. The performance in the deadlift could possibly correlate with performance in a TFM event in which a heavier load is carried. Additionally, it has been suggested that upper body strength is more important for a TFM compared to lower body strength [22,46,52], so this may have also influenced the results from this study.

The standing power throw provides a measure of overall total-body power and coordination [58,59,60]. There has been limited research on how power relates to TFM performance. A study of specialist police undertaking three 5-km load carriage marches as fast as possible with a 25-kg load found that lower body power, as measured by a 10-m sprint, was significantly correlated with the first march (*r* = 0.373, *p* < 0.05) but not the two subsequent marches (*r* = 0.178 and 0.217, *p* > 0.05) [44]. It could be that a short, one-time expression of power that is needed for the standing power throw is not as relevant to the extended, long-duration effort required for the TFM. Power, as measured by the standing power throw, may be more important for other soldier tasks that involve actions such as jumping, sprinting, or throwing [3,8,57,61]. In agreement, in the present study cadet sample performance in the standing power throw test was not related to the TFM. Regardless, cadets had a mean score of approximately 74 out of 100 points or 8 m, indicating that many cadets in this sample could stand to improve their power production.

Maximal push-ups provide a measure of the muscular endurance [62,63,64,65]. The push up as performed in this study by the cadets was not significantly correlated to the TFM. While upper body muscular endurance can contribute to the TFM [22], it could be that the push-up movement and involved musculature are not related to load carriage and the type of movements performed in the TFM by this sample of cadets. Muscular endurance, as measured by the push-up, may be more important for other soldier tasks that involve repeated movements, such as movement over obstacles, constructing a fighting position, etc. [3,7,10]. In the push-up event, cadets did relatively well, with a mean score of approximately 86 out of 100 points or 46 repetitions. It is likely that cadets averaged higher on this event over all others because the APFT had a push-up event, so it has traditionally been an often-utilized exercise in the Army [13,66]. It is possible that the cadets had much more experience with the push-up exercise, which also contributed to their performance.

This study has certain limitations that should be acknowledged. Only one university ROTC program was analyzed in this study; cadets from other university ROTC programs may demonstrate different fitness parameters. The sample size of the data provided by ROTC was small (*N* = 29) and consisted mostly of MSI and MSII cadets. Accordingly, the results should be considered with the sample size in mind. To provide some background, the ROTC cadre had originally agreed to provide the investigators with data for the entire battalion. However, training plans were altered, and not all cadets completed an individual TFM assessment, which affected the dataset provided to the researchers. Forthcoming studies should replicate this research, with a greater sample size and more MSIII and MSIV cadets. Nonetheless, given there is limited research on the association between fitness parameters and TFM performance in ROTC cadets, the results from this study are still valuable. Future studies should analyze these associations in active-duty combat arms professions of the Army as relationships may differ. In the fitness testing data provided by ROTC cadre, male cadets outnumbered female cadets more than two to one, thus making the sample size of females relatively small. Nonetheless, this is typical of military populations, where there tends to be a greater number of males relative to females [67]. Only data from one TFM was analyzed; it was shorter than usual, and cadets carried a type of load that was lighter than usual [20]. A different TFM test could provide different results, and higher intensity TFM (longer distance, heavier load, etc.) should be investigated in ROTC cadets.

## 5. Conclusions

Male cadets in this study, in general, scored significantly higher than female cadets, in general, in ACFT total score and all events except push-ups, with no significant difference between the sexes in TFM performance. Even though further analysis is required, based on data from this sample of ROTC cadets, changing the ACFT scoring standards to a sex- and aged-based scale could mask deficiencies in fitness between the sexes. ACFT total score and both raw and scaled scores for leg tuck, 2-mile run, and sprint-drag-carry showed significant moderate-to-large associations with TFM. Within the context of the study limitations, the results from this study suggested that anaerobic capacity, upper body and trunk strength, and aerobic endurance are fitness parameters associated with TFM performance in ROTC cadets. Cadets that need to improve TFM performance should consider the development of upper body strength and anaerobic and aerobic capacity within their fitness training. This could be achieved with a periodized concurrent resistance training and running program that also includes anaerobic conditioning and foot marching.

## Figures and Tables

**Table 1:** Descriptive data (mean ± SD) for the Army Combat Fitness Test event (3-repetition maximum deadlift (deadlift), standing power throw, hand-release push-ups (push-ups), sprint-drag-carry, leg tuck, 2-mile run) raw scores and tactical foot march score for the overall group, males, and females.

**Assessment**	**Overall** **(*N* = 29)**	**Males** **(*n* = 20)**	**Females** **(*n* = 9)**	***p***	***d***	***d*** **Strength**
Deadlift (kg)	106.19 ± 31.15	119.17 ± 27.83	77.34 ± 14.10 *	<0.001	1.70	Large
Standing Power Throw (m)	8.04 ± 2.24	9.05 ± 1.92	5.80 ± 0.77 *	<0.001	1.95	Large
Push-ups (repetitions)	46.10 ± 10.15	48.10 ± 9.11	41.67 ± 11.47	0.058	0.65	Moderate
Sprint-Drag-Carry (s)	127.41 ± 22.80	118.15 ± 19.46	148.00 ± 15.16 *	<0.001	1.51	Large
Leg tuck (repetitions)	10.20 ± 6.58	11.89 ± 6.44	5.86 ± 5.01 *	0.037	0.99	Moderate
2-mile Run (min:sec)	17:36 ± 2:31	16:55 ± 2:32	19:00 ± 1:55 *	0.039	1.02	Moderate
TFM (min)	64.66 ± 7.21	64.90 ± 7.31	64.11 ± 7.41	0.791	0.11	Trivial

* Significantly (*p* < 0.05) different from the male cadets.

**Table 2:** Descriptive data (mean ± SD) for the Army Combat Fitness Test event (3-repetition maximum deadlift (deadlift), standing power throw, hand-release push-ups (push-ups), sprint-drag-carry, leg tuck, 2-mile run) scaled and total point scores for the overall group, males, and females.

**Assessment**	**Overall** **(*N* = 29)**	**Males** **(*n* = 20)**	**Females** **(*n* = 9)**	***p***	***d***	***d*** **Strength**
Deadlift	77.34 ± 13.54	82.85 ± 12.51	65.11 ± 5.04 *	<0.001	1.64	Large
Standing Power Throw	73.69 ± 11.45	78.60 ± 10.47	62.78 ± 1.99 *	<0.001	1.79	Large
Push-ups	86.21 ± 9.94	88.15 ± 8.99	81.89 ± 11.12	0.118	0.65	Moderate
Sprint-Drag-Carry	78.17 ± 13.84	83.80 ± 12.92	65.67 ± 4.36 *	<0.001	1.56	Large
Leg Tuck	80.16 ± 13.49	83.72 ± 12.97	71.00 ± 10.72 *	0.031	1.02	Moderate
2-mile Run	74.29 ± 11.67	77.63 ± 12.33	67.22 ± 5.89 *	0.006	1.00	Moderate
Total	457.62 ± 75.22	487.35 ± 60.91	391.56 ± 62.23 *	<0.001	1.56	Large

* Significantly (*p* < 0.05) different from the male cadets.

**Table 3:** Partial correlations (controlling for sex) with the tactical foot march and Army Combat Fitness Test event (3-repetition maximum deadlift (deadlift), standing power throw, hand-release push-ups (push-ups), sprint-drag-carry, leg tuck, 2-mile run) raw scores in ROTC cadets (*N* = 29).

**Assessment**	***r***	***p***	***r*** **Strength**
Deadlift	−0.102	0.644	Small
Standing Power Throw	0.021	0.925	Small
Push-ups	−0.366	0.086	Moderate
Sprint-Drag-Carry	0.503 *	0.014	Large
Leg Tuck	−0.512 *	0.013	Large
2-mile Run	0.506 *	0.014	Large

* Significantly (*p* < 0.05) correlated with the TFM.

**Table 4:** Partial correlations (controlling for sex) with tactical foot march and Army Combat Fitness Test event (3-repetition maximum deadlift (deadlift), standing power throw, hand-release push-ups (push-ups), sprint-drag-carry, leg tuck, 2-mile run, scaled scores and total score in ROTC cadets (*N* = 29).

**Assessment**	***r***	***p***	***r*** **Strength**
Deadlift	−0.044	0.843	Small
Standing Power Throw	0.004	0.986	Small
Push-ups	−0.372	0.081	Moderate
Sprint-Drag-Carry	−0.463 *	0.026	Moderate
Leg tuck	−0.516 *	0.012	Large
2-mile Run	−0.531 *	0.009	Large
Total	−0.528 *	0.004	Large

* Significantly (*p* < 0.05) correlated with the TFM.

## Data Availability

Restrictions apply to the availability of these data due to ethical, legal, and privacy concerns.
